# 抗血管药物治疗放射性脑坏死专家共识

**DOI:** 10.3779/j.issn.1009-3419.2022.101.18

**Published:** 2022-05-20

**Authors:** 意 陈, 鑫 王, 冰 孙, 茂斌 孟, 恩敏 王, 智勇 袁, 洪卿 庄, 中国医药教育协会肿瘤放射治疗委员会

**Affiliations:** 1 100191 北京，北京大学第三医院肿瘤放疗科 Department of Radiation Oncology, Peking University Third Hospital, Beijing 100191, China; 2 200000 上海，复旦大学附属华山医院肿瘤放疗科 Department of Radiation Oncology, Fudan University Huashan Hospital, Shanghai 200000, China; 3 100000 北京，解放军总医院第五医学中心肿瘤放疗科 Department of Radiation Oncology, The Fifth Medical Center of PLA General Hospital, Beijing 100000, China; 4 300000 天津，天津医科大学肿瘤医院肿瘤放疗科 Department of Radiation Oncology, Tianjin Medical University Cancer Institute and Hospital, Tianjin 300000, China

**Keywords:** 放射性脑坏死, 抗血管生成药物, 贝伐珠单抗, 专家共识, Radioactive brain necrosis, Antiangiogenic drugs, Bevacizumab, Expert consensus

## Abstract

血管内皮生长因子（vascular endothelial growth factor, VEGF）的高水平表达是放射性脑坏死（cerebral radiation necrosis, CRN）发生的重要机制。抗血管生成药物（贝伐珠单抗）通过抑制VEGF，作用于脑坏死区域周围的血管组织，减轻CRN引起的脑水肿。许多研究证实贝伐珠单抗可有效缓解脑坏死症状，改善患者的体力状况评分以及减轻影像学上脑水肿范围。抗血管治疗的疗效主要与药物作用时长相关，低剂量抗血管药物即可达到较好的治疗效果。预防是最好的治疗，CRN的发生与肿瘤相关因素和治疗相关因素相关，通过控制两方面因素，可有效预防CRN。

## Abstract (en)

Vascular damage is followed by vascular endothelial growth factor (VEGF) expression at high levels, which is an important mechanism for cerebral radiation necrosis (CRN) development. Antiangiogenic agents (Bevacizumab) alleviates brain edema symptoms caused by CRN through inhibiting VEGF and acting on vascular tissue around the brain necrosis area. Many studies have confirmed that Bevacizumab effectively relieves symptoms caused by brain necrosis, improves patients' performance status and brain necrosis imaging. Considering that the efficacy of antiangiogenic therapy is mainly related to the duration of drug action, low-dose antiangiogenic agents can achieve favorable efficacy. Prevention is the best treatment. The occurrence of CRN is associated with tumor-related factors and treatment-related factors. By controlling these factors, CRN can be effectively prevented.

## 1 序言

随着立体定向放疗在脑部病灶中的应用越来越广泛，放射性脑坏死（cerebral radiation necrosis, CRN）成为立体定向放疗后远期第一并发症^[1, 2]^。然而，CRN的发生机制，治疗，预防等方面临床还有很多需要进一步规范的空间，因此，我们邀请了在这一领域具有丰富经验的中国专家审议上述重要问题并初步协商达成一致意见。本共识旨在为抗血管生成药物治疗CRN提供详实的临床依据并初步规范我国抗血管药物治疗CRN的临床应用。

## 2 方法学

本共识撰写的第一步是由共识专家组组长与执笔作者基于已发表的临床研究证据，并结合临床经验，整理出共识初稿。之后由7名指南撰写专家组成员经过3轮专家组会议对共识初稿内容进行讨论和修改，最终确定共识内容。因此，本共识提出的推荐内容是基于现有的中国和国际临床高质量循证医学证据以及专家组广泛认可的临床经验。

## 3 共识

### 3.1 共识一

抗血管生成药物治疗放射性脑坏死的机制在许多关于CRN发生和发展的理论中，血管机制理论被广泛接受。由于辐射对肿瘤周围血管组织的影响，导致血管组织损伤，组织和血管之间的氧扩散紊乱，进而导致组织缺氧，从而引发缺氧诱导因子（hypoxia-inducible factor, HIF）-1α表达增加。其次，肿瘤组织缺氧和HIF-1α表达升高刺激反应性星形胶质细胞分泌促血管生成因子（vascular endothelial growth factor, VEGF）。VEGF高水平表达导致异常新生血管形成，该新生血管结构紊乱脆弱，通透性高，促进周围组织渗出，从而导致脑水肿。水肿又引起局部组织缺血缺氧，最终发展为CRN^[1-4]^。抗血管生成药物，目前主要指贝伐珠单抗，它是一种重组人单克隆抗体，可与VEGF结合，防止VEGF与其受体（Flt-1和KDR）在内皮细胞表面结合，起到修剪血管、调节血管通透性、减轻脑坏死引起的脑水肿和治疗脑坏死的作用^[2]^。

### 3.2 共识二

抗血管生成药物治疗放射性脑坏死的疗效①抗血管药物治疗放射性脑坏死的疗效：2007年，Gonzalez等^[5]^首次报道贝伐珠单抗治疗CRN的疗效，尽管样本量小，但仍是一项重要的开创性研究。此后约有几十项关于贝伐珠单抗治疗CRN的研究发表。但是，CRN的临床研究不同于癌症治疗的研究，因为CRN是一种不良反应，在临床治疗中应尽量减少其发生率，因此，大多数研究纳入病例数较少。目前约有4项前瞻性对照临床研究^[6-9]^和4项前瞻性单臂临床研究^[10-13]^以及多项回顾性临床研究^[14]^。这些研究结果表明，贝伐珠单抗能显著改善发生CRN的患者的脑水肿、症状和Karnofsky体力状况（Karnofsky performance status, KPS）评分，有效率为54.9%-100%；此外，其副作用相对较轻，≥3级的副作用相对少见。②抗血管药物治疗放射性脑坏死剂量选择：根据临床试验提供的证据，贝伐珠单抗的剂量通常约为5 mg/kg, *q2-3w*，患者至少接受2个疗程的治疗，最大疗程数目前没有定论。值得注意的是，2019年庄洪卿等学者团队^[10]^通过一项前瞻性II期临床研究探索了极低剂量贝伐珠单抗（1 mg/kg/3周）治疗CRN，症状缓解率高达90%，影像学缓解率高达95.2%，毒副反应相比高剂量显著降低，此研究表明即使极低剂量的贝伐单抗治疗CRN的效果仍然较好，且毒副反应更低。另一项研究^[15]^也得到了类似的结论。因此，我们推荐贝伐珠单抗治疗CRN的剂量为≤5 mg/kg，*q2-3w*。③抗血管药物治疗放射性脑坏死适应证：与肿瘤治疗不同，CRN治疗的目标不是延长生存期，而是减少症状和改善生活质量。考虑到贝伐珠单抗的治疗目标，使用贝伐珠单抗的关键指征是治疗CRN导致的症状。因此，庄洪卿等研究团队^[2, 16]^通过研究建议只治疗有症状的患者，监测无症状的患者。

### 3.3 共识三

放射性脑坏死的预防 预防是CRN最好的治疗。CRN的发生与多种风险因素相关，充分控制这些风险因素，能够显著降低CRN的发生率。①肿瘤相关风险因素：目前的证据显示，CRN的发生风险与脑转移肿瘤的大小、组织学类型、基因型和异质性指数相关。Blonigen等^[17]^报道接受治疗的脑转移大小与CRN显著相关。Miller等^[18]^发现脑放射性坏死与病灶大小、肾和非小细胞肺腺癌以及异质性指数之间存在显著统计学意义的相关性；此外，亚组分析发现人表皮生长因子受体2（human epidermal growth factor receptor 2, *HER-2*）扩增、*BRAF* V600+突变状态和间变性淋巴瘤激酶（anaplastic lymphoma kinase, *ALK*）重排均与CRN显著相关；这项研究代表了关于肿瘤生物学和脑放射性坏死之间的联系的第一个证据，并具有潜在的临床意义。最近的全基因组研究^[19]^显示，在胶质瘤细胞株U87中，Cep128的突变失活与颞叶辐射损伤的风险相关；这是第一个涉及辐射损伤敏感基因的研究，为辐射诱发脑损伤的潜在机制提供了新的见解。综上，如果肿瘤具有特异基因改变，可能导致了CRN发生率增加，预防主要是通过调整治疗相关参数。②放疗相关风险因素：放疗剂量、单次分割剂量大小、分割次数以及是否联合全脑放疗均是影响CRN的风险因素。研究表明放疗剂量越高，CRN的发生风险越大。庄洪卿等团队^[20]^发现当立体定向放射外科治疗（stereotactic radiosurgery, SRS）的生物等效剂量 > 74.1 Gy时（分割次数1次-5次），发生CRN的风险显著升高。另一项研究指出V10 Gy-16 Gy是接受SRS治疗的患者是否发生CRN的显著预后因子（*P* < 0.001），当V10 Gy > 12.6 mL以及V12 Gy > 10.9 mL时，CRN风险达到47%^[21]^。有研究报道强调放疗时，50 Gy/25 f是CRN发生风险的剂量阈值，低于这个阈值，CRN的发生风险被认为是最小的^[22]^。研究^[23]^显示单次分割剂量越高，发生CRN的风险越大，正如在一项研究中所报道的，2 Gy/f（总剂量60 Gy）发生CRN的风险大于1.7 Gy/f（总剂量60 Gy）。庄洪卿等^[20]^学者报道，生物等效剂量相当时，SRS的分割次数≤2时，CRN的发生率显著升高。另一项研究^[24]^报道V12 Gy与症状性CRN显著相关，V12 Gy≤5 cc、5 cc-10 cc、10 cc-15 cc以及≥15 cc时，CRN发生率分别为23%、20%、54%和57%。研究^[20, 25]^显示接受SRS联合全脑放疗的脑肿瘤患者发生CRN的风险显著升高。综上，控制放疗剂量，提高分割次数，避免联合全脑放疗能够有效预防CRN的发生。③药物治疗相关风险因素：研究^[1]^表明，放疗联合药物治疗（化疗、靶向治疗和免疫治疗）可增加CRN发生的风险。Cagney等^[26]^报道，放疗联用培美曲塞显著增加了CRN的发生率。Patel等^[27]^报道接受BRAFi和SRS治疗的患者中CRN发生率高达22%，而仅接受SRS的患者中CRN发生率仅有11%。有研究^[28]^发现同时接受免疫检查点抑制剂（immune checkpoint inhibitors, ICIs）和SRS治疗的黑色素瘤脑转移的患者与未接受ICIs治疗的患者相比，前者发生CRN的风险更高，且总生存期更长。还有一项研究^[29]^发现，在接受伽玛刀放疗的患者中，与接受化疗相比，使用ICIs与更高的CRN发生率有关。然而，值得注意的是，庄洪卿等^[30]^报道酪氨酸激酶抑制剂（tyrosine kinase inhibitor, TKI）耐药后再行脑部转移SABR比耐药前放射性脑坏死风险增加3倍。

综上，行脑肿瘤放疗时减少化疗、靶向或免疫药物的同时使用，可以有效减少CRN的发生，从而起到预防CRN的作用。如果靶向药物必须使用时，尽可能在靶向药物出现耐药前行SRS能够预防一部分患者CRN的发生。

## 4 总结和展望

VEGF的高水平表达是CRN发生的重要机制。抗血管生成药物（贝伐珠单抗）通过抑制VEGF发挥治疗CRN的作用。目前研究证实贝伐珠单抗治疗CRN的疗效确切，副反应较小。CRN的发生与多种因素相关，控制相关因素，做好预防是关键。然而，抗血管生成药物治疗CRN的长期疗效、适应证、给药方式和剂量的优化以及耐药机制等诸多问题未来还需要更多的临床研究探索和阐明。
